# Sentinel Node Biopsy in the Neck Management of cN0 Sinonasal Squamous Cell Carcinoma: A Multicenter Pilot Trial on Safety and Feasibility

**DOI:** 10.1245/s10434-026-19896-4

**Published:** 2026-06-02

**Authors:** Johannes Doescher, Adrian von Witzleben, Nina Eberhardt, Christina Sauter, Caroline Mlynarcik, Alexandra Peiper, Eric Treutlein, Patrick J. Schuler, Fabian Sommer, Simon Laban, Ambros J. Beer, Johannes Zenk, Thomas K. Hoffmann

**Affiliations:** 1 Department of Otorhinolaryngology and Head & Neck Surgery, University Medical Center Ulm, Ulm, Germany; 2 Department of Otorhinolaryngology, University Hospital Augsburg, Augsburg, Germany; 3 Department of Nuclear Medicine, University Hospital Ulm, Ulm, Germany; 4 Department of Otorhinolaryngology, Head and Neck Surgery, University Hospital Heidelberg, Heidelberg, Germany; 5 Department of Otorhinolaryngology, Academic Teaching Hospital Feldkirch, Feldkirch, Austria

**Keywords:** Sinonasal squamous cell carcinoma, Sentinel node biopsy, cN0 neck, SPECT/CT, Gamma probe

## Abstract

**Background:**

To date, there are no clear recommendations for the treatment of a clinically inconspicuous neck (cN0) in sinonasal squamous cell carcinoma. Elective neck dissection or neck irradiation appears too aggressive given the relatively low occult metastasis rates. However, the development of neck lymph node metastases is significantly associated with worse survival, therefore patients at relevant risk need to be identified. The aim of this trial was to evaluate feasibility and safety of sentinel node biopsy for sinonasal squamous cell carcinoma.

**Patients and Methods:**

This was a prospective, single-arm, open label, multicentric pilot trial (phase II) designed to evaluate the safety and feasibility of sentinel node biopsy (SNB) of patients with sinonasal squamous cell carcinoma and clinical N0 status. 24 h before surgery, radiocolloids were injected around the tumor and lymphoscintigraphy with single-photon emission computed tomography (SPECT)/computed tomography (CT) was performed. After resection of the primary tumor, the sentinel lymph node was identified using a gamma probe and resected via minimal invasive incision.

**Results:**

At least one sentinel node could be detected in each of the 22 patients, predominantly in level Ib and IIa. The average lymph node yield after SNB was two, and 122.7% relative to the marked lymph nodes. SNB identified two cases (T2 and T4a) with micrometastases resulting in an occult metastasis rate of 9.1%. The procedure was safe and aesthetically satisfactory.

**Conclusions:**

Sentinel node biopsy for sinonasal squamous cell carcinoma is a feasible and safe procedure to detect occult metastases and identify patients at risk for nodal relapse. The results of the study encourage confirmatory, randomized trials.

**Electronic supplementary material:**

The online version of this article (https://doi.org/10.1245/s10434-026-19896-4) contains supplementary material, which is available to authorized users.

Sinonasal carcinomas are rare tumors embracing a variety of entities. The incidence is approximately 0.5 to 1 newly diagnosed case per 100,000.^1^ The most common entity is squamous cell carcinoma (SNSCC), followed by adenoid cystic carcinoma (SNACC) and adenocarcinomas (SNAC).^2,3^

There are three groups in terms of prognosis: esthesioneuroblastoma and SNACC with a more favorable prognosis, SNSCC and SNAC with an intermediate prognosis, and those with a relatively poor prognosis (melanoma and undifferentiated carcinoma). An improvement of survival could only be seen for SNAC in the last decades.^4^

Survival is dependent on the primary site and regional metastases. Nodal involvement has been shown to worsen 5-year overall survival (OS) severely.^5,6^ The reported frequencies of lymph node metastasis differ widely in the literature. These range from 3.3 up to 26% at primary diagnosis with reports of high neck relapse rates with up to 33% of untreated necks.^7,8^ In our own studies on SNSCC, primary lymph node metastases were found in 4.5% of cases and lymph node relapse occurred in 11.4%.^9^ Occult metastasis is reported to be found in 3–25% of sinonasal malignancies depending on the neck treatment.^7,10–12^ Risk factors for developing lymph node metastases are discussed broadly and almost every study on this subject comes to a different conclusion and recommendation for the treatment of the clinical N0 neck.^8^ A more recent study sees the risk of developing lymph node metastases not depending on T stage, but rather on invasion of distinct structures such as the dura.^13^ This was also confirmed for advanced T stages, where an omitted elective neck treatment did not lead to a higher percentage of nodal recurrence.^14^ Another study examined T3 and T4 stage SNSCC only and did not find an advantage for survival after elective neck dissection (END) of clinically inconspicuous neck (cN0) cases.^15^

The rarity of these tumors and the different reports about lymph node involvement have made it difficult to establish a gold standard for neck management in the past. There are several recommendations in the literature but the clinically N0 neck is usually not recommended to be treated even in advanced disease, or it is a surgeons decision on a case to case basis.^5,7,16,17^ A reliable, feasible and safe method for the revelation of occult metastases would be desirable.^18^ Sentinel node biopsy (SNB), which is used for several entities, including early oral squamous cell carcinoma, represents an option. The concept of SNB has been long established in other tumor entities since the 1990s. A sentinel node (SN) is defined as the first lymph node in the lymphatic drainage of the primary tumor. There are different techniques in tracing the respective node with lymphoscintigraphy using dyes and radioisotopes. In the head and neck area several trials exist showing a high SN identification rate of 97.7–100% and a low false negative rate of 3.3%, if at least three SN were resected.^19,20^ For sinonasal carcinoma a few case reports have been published over the last decade, either being limited in case numbers or of retrospective design, but with promising results.^12,21^

This multicenter study examines the use of sentinel lymph node biopsy for detecting occult lymph node metastases in patients with cN0 sinonasal squamous cell carcinoma, with the aim of testing its safety and feasibility.

## Patients and Methods

### Trial Design

The study was set up as prospective, single-arm, open label, multicentric pilot trial (phase II) designed to evaluate the safety and feasibility of sentinel node biopsy of patients with sinonasal squamous cell carcinoma and clinical N0 status.

Sample size was determined via a time-driven design. A recruitment period of 18 months was determined. Owing to institutional experiences of SNSCC incidences, the recruitment of approximately 25 patients was expected within this period. Per protocol, recruitment could be prolonged if reasonable factors (e.g., pandemic-related restrictions) occurred. In this case, the study was due to be terminated at 20 enrolled patients for final analysis. Patients continued to be assessed in routine oncological follow-ups every 3 months for the first 2.5 years and every 6 months for the following 2.5 years.

### Participants

This multicenter study was conducted at two tertiary referral hospitals. Prior to enrollment, each patient signed an informed consent. The study procedures were approved by the Institutional Review Board and performed in accordance with the ethical standards of the 1975 Declaration of Helsinki. This study was registered with the German Clinical Trial Registry (DRKS00022304).

Patients with a histologically proven squamous cell carcinoma of the nasal vestibulum and the sinonasal tract of any tumor size (cT1-cT4a) without clinically suspect lymph nodes (assessed at least by ultrasound), who were recommended for curative tumor resection by a multidisciplinary tumor board, were eligible for inclusion in the study. Further inclusion criteria were age ≥ 18 years, signed informed consent, ECOG PS ≤ 2 / Karnofsky PS ≥ 60%. Main exclusion criteria were a history of a prior head and neck malignancy (except for completely resected basal cell carcinoma), a history of treatment of the neck of any cause, an entity other than squamous cell carcinoma and clinically suspect lymph nodes or distant metastases.

### Intervention and Outcome Measures

After enrollment, patients were scheduled for resection of their primary tumor and SNB. Preoperative lymphoscintigraphy with radiocolloids (Technetium-99m human serum albumin nanocolloid, particles ≤ 80 nm, mean 110–140 MBq, in four subcutaneous injections) was performed within 24 h before surgery. SNs were visualized by planar scintigraphy (whole body scan in anterior projection, 30 cm/min) using a Symbia T2 single-photon emission computed tomography (SPECT)/computed tomography (CT) or a Symbia Pro.specta X3 SPECT/CT gamma camera system (both Siemens Healthineers, Erlangen, Germany) with additional SPECT/CT with unenhanced low-dose CT (120 kV, 15 mA, 25 effective mAs, attenuation-corrected SPECT/CT reconstructions in three planes) of the suspected neck area. Additionally, marked SNs were assessed by ultrasound before surgery. After resection of the primary tumor, gamma probe guided SNB was performed in the same surgery. In case of positive lymph nodes after SNB, a two-stage complementary neck dissection was conducted. If necessary and feasible, this was combined with a two -stage resection of the primary, if pathological results showed positive or close margins. Adjuvant radio- or chemoradiotherapy was recommended on the basis of pathology reports and imaging by a multidisciplinary tumor board (MTB) decision.

Core objectives were the safety and feasibility of sentinel node biopsy for clinically lymph node negative resectable sinonasal carcinomas.

Safety was measured by the frequency of severe adverse events. Adverse events of special interest were defined by damage to the facial nerve and heavy scarring. Damage to the facial nerve was measured by House–Brackmann score (HBS). Scarring was measured using the Patient Observer Scar Assessment Scale 2.0 (POSAS) by the patient and the surgeon. The questionnaire consists of six questions, with a maximum score of ten representing the worst scar imaginable. A total score of 60 points is possible if all questions are answered with a score of 10, representing the worst possible outcome.

Feasibility was investigated by the number of harvested SNL and the detection rate of occult metastases.

Overall and metastases free survival was evaluated as a side objective in this patient cohort. Endpoints for the objective were time to development of distant and/or regional metastases and death from any cause.

### Statistics

All data were analyzed descriptively. Continuous variables are reported as mean with standard deviation (SD) or median with minimal to maximal value. Qualitative features were calculated as absolute and relative rates. Graphic depiction was done with Prism (Graphpad). The core objective “safety” is described as absolute number and percentual rate of median HBS results. POSAS results are reported as mean score value and SD. Detection rates of occult metastases were put in relation to harvested sentinel lymph nodes in absolute and relative numbers with 95% confidence interval (CI). The secondary endpoints “overall survival,” “regional metastasis-free survival,” and “distant metastasis-free survival” were calculated using Kaplan–Meier analysis. A *p*-value < 0.05 was considered significant.

## Results

### Participants

**Fig. 1:**
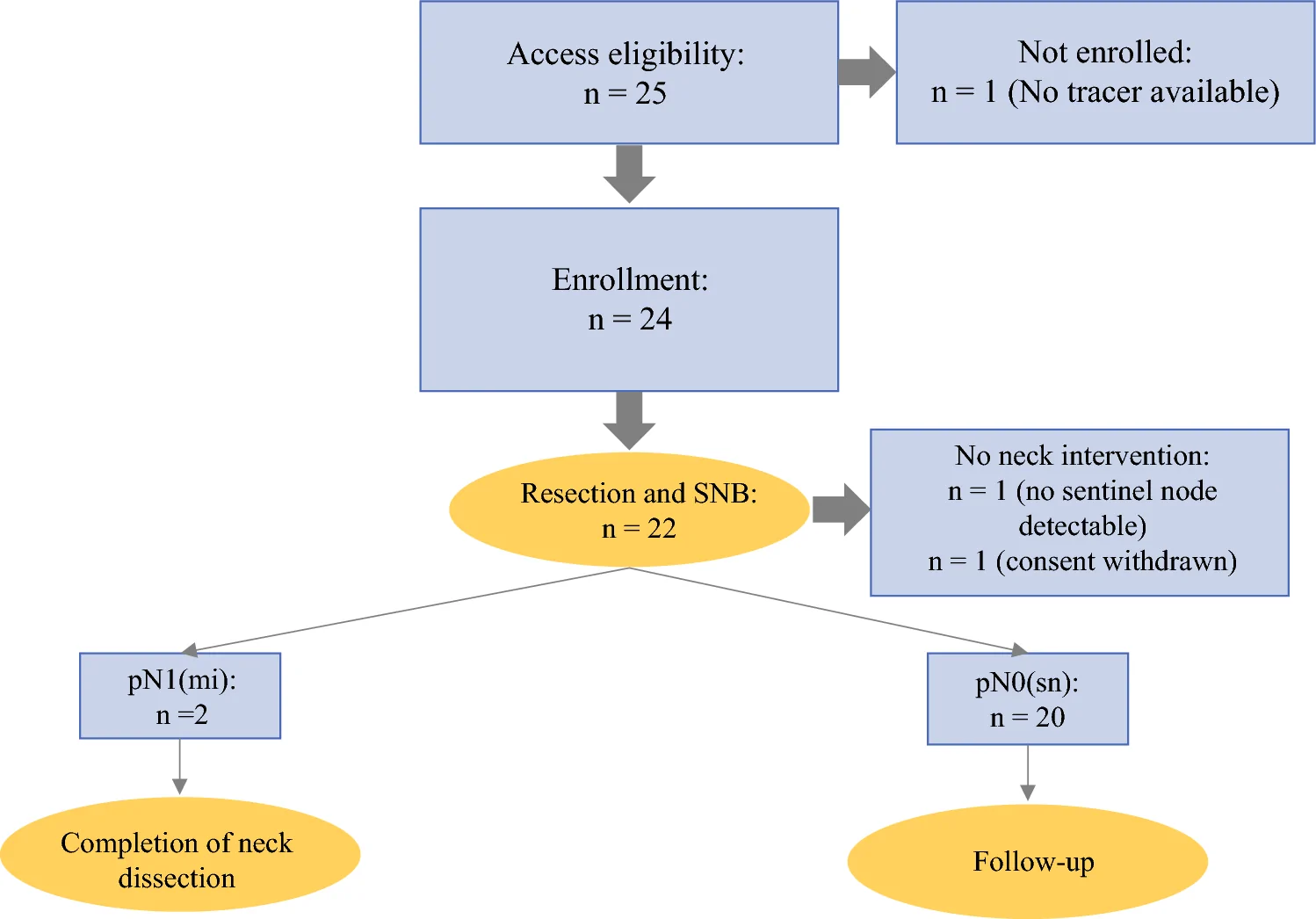
Consort diagram displaying patients’ flow in study

A total of 24 patients gave their consent and were enrolled in the study from August 2021 until July 2025. Of those, 22 patients were treated by tumor resection of the primary and SNB. Two patients had to be excluded after enrollment. In one patient no sentinel node could be detected despite repeated tracer injection and SPECT-CTs. The other patient withdrew consent shortly before the intervention and opted for definitive chemoradiotherapy. All treated patients were included in the efficacy and safety analyses (*n* = 22). Figure 1 provides a consort diagram of the study population.

### Clinical Characteristics

A summary of clinical characteristics is presented in Table 1. Patients’ median age at diagnosis was 67.5 years, and there were slightly more female patients (59.1%). All patients suffered from squamous cell carcinoma occurring in the nasal cavity. All cases were staged cN0 by ultrasound and extended imaging by CT or magnetic resonance imaging (MRI) scan. T stages were evenly distributed within the stages T1, T2, and T4.

**Table 1:** Clinical parameter of evaluable patients

Clinical parameter	Number of patients *n* = 22
Median Age (range)	67.5 (33–81) years
Sex	
Male	9 (40.9%)
Female	13 (59.1%)
Ethnicity	
White	22 (100%)
Tumor site	
Nasal cavity	22 (100%)
T stage (pathological)	
T1	6 (27.3%)
T2	8 (36.4%)
T3	1 (4.5%)
T4a	7 (31.8%)
N stage (pathological)	
N0(sn)	20 (90.9%)
N1(mi)	2 (9.1%)
N1-3	0
M stage	
M0	22 (100%)

### Sentinel Node Biopsy

**Fig. 2:**
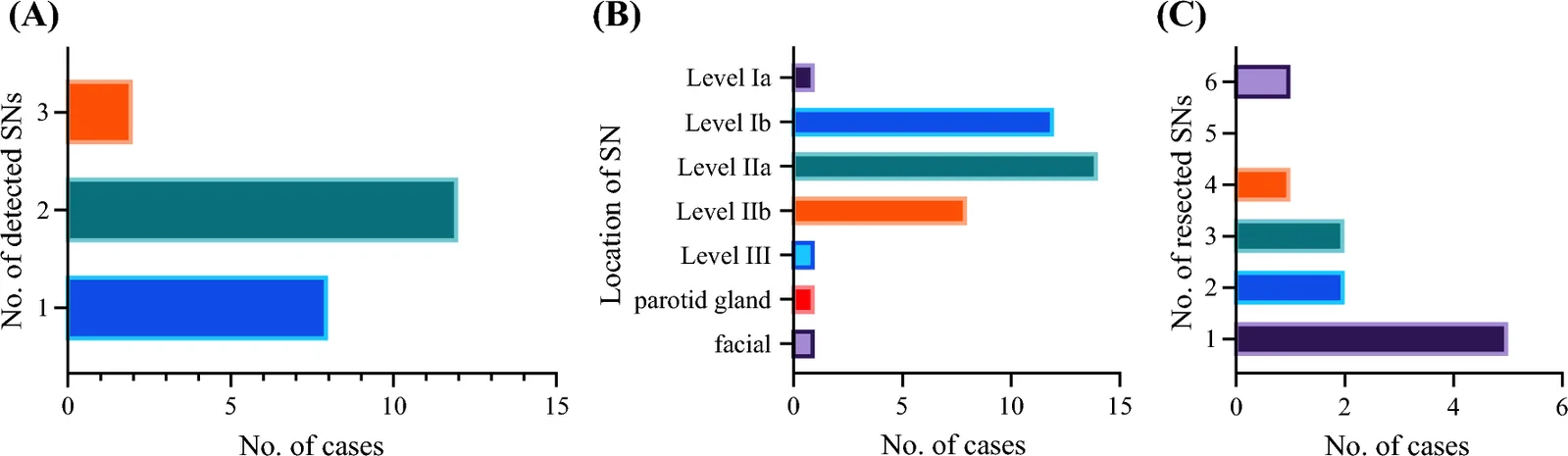
Bar plots showing **A** number of detected SNs by lymphoscintigraphy, **B** distribution of location of SNs, **C** number of resected LNs

Except for one patient, at least one SN could be detected by SPECT-CT imaging. In detail, a single node could be detected in 8 patients, two nodes in 12 patients, and three nodes in another 2 patients (Fig. 2a). SNs were mostly apparent in level Ib and IIa, followed by IIb. Rarely, SNs were found to be in level Ia, III, the parotid gland or facial groups (Fig. 2b). More than 50% of the patients had bilateral SNs (*n* = 12), 10 only had ipsilateral SNs.

After resection of the primary, an average of two lymph nodes per patient could be harvested (range 1–6; Fig. 2c). In relation to the number of marked LNs, the LN yield was 122.7% (95% CI 93.12,–152.33). SNB led to the identification of a micrometastasis in two cases (9.1%, one pT2, one pT4a). Both patients underwent completion of neck dissection with no further evidence of nodal metastases.

### Oncological Outcome

Median follow-up time was 24.5 months (range 2–51 months). Apart from two cases, there were no recurrences and/or distant metastases observed in the follow-up period. In the two cases of recurrence, these occurred after 5 and 8 months, respectively. Interestingly, one case with recurrent disease, both local and nodal, was one of the two cases with positive SNB (pT2). Recurrence occurred despite completion of ND. No adjuvant radiotherapy was recommended by MDT because of no further tumorous LNs. The other patient (pT4a) developed pulmonal metastases after refusing adjuvant RT and is currently under palliative immunotherapy. All patients are still alive, and no further recurrences have been reported.

### Safety

**Fig. 3:**
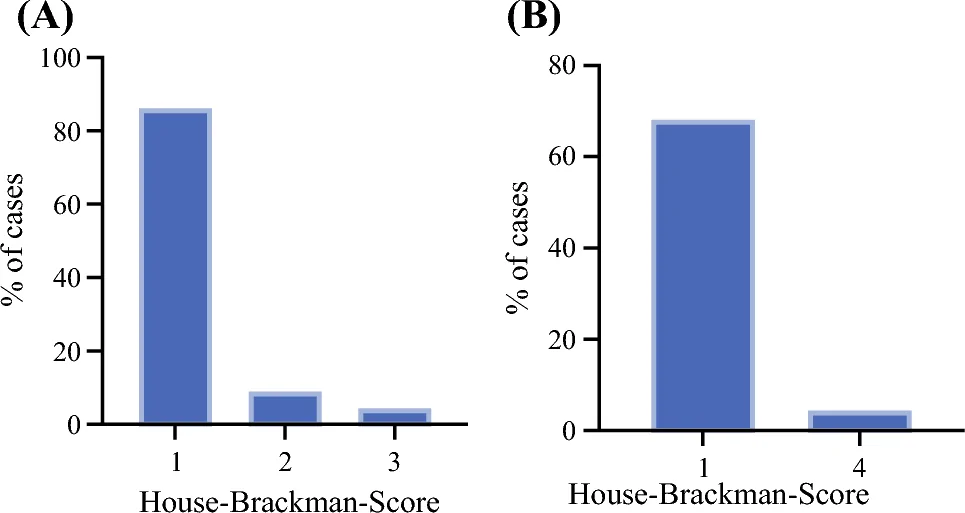
Bar plots show the distribution of House–Brackman score **A** 3 weeks postoperatively and **B** 3 weeks after surgery

Safety assessments were obtained 2 weeks postoperatively as well as 3 months later. There were no AEs related to SNB, except for facial nerve impairment. Slight facial nerve palsy (House–Brackman score 2) in two cases resolved within 3 months, whereas in one case with moderate involvement (House–Brackman score 3), recovery took 12 months after temporary progression to score 4 (Fig. 3).

### Aesthetics

**Fig. 4:**
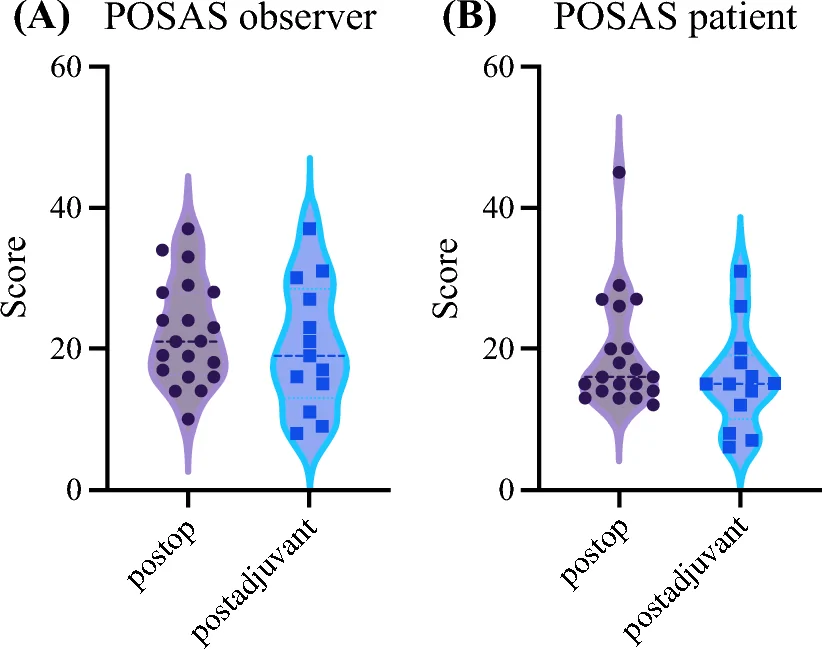
Violin plots show overall results of aesthetic assessment using the POSAS questionnaire

The aesthetic outcome was assessed by both medical professionals and patients. Observers scored SNB scars with a mean postoperative score of 22.25/60 (SD ± 7.34) and a mean score after 3 months of 20.31/60 (SD ± 8.97) (Fig. 4a). Patients assessed their own scars with a mean postoperative score of 19.25/60 (SD ± 8.05), which improved further after 3 months to 15.62/60 (SD ± 7.14) (Fig. 4b). The mean values ± SD for each item are presented in Table S1 and S2.

## Discussion

This is the first controlled, prospective trial investigating the application of sentinel node biopsy in sinonasal squamous cell carcinoma. Using SNB, micrometastases were detected in 2 of 22 patients, resulting in an occult metastasis detection rate of 9.1%. After several case reports on the theoretical feasibility of SNB in sinonasal carcinoma, a preliminary study first demonstrated its practical applicability in six cases, reported a sentinel node detection rate of 66.6%, and identified one occult metastasis.^12^ More recently, a retrospective analysis of 20 cases was published. This cohort included a variety of histologies, with only 10 squamous cell carcinomas, and 70% of tumors classified as T1/T2. Although at least one sentinel node was successfully resected in all cases, the exact number per patient was not reported. As in the present study, sentinel nodes were mainly located in Levels I and II, though these levels were not further differentiated into sublevels a or b. Ultimately, no occult metastases were detected.^21^ Several factors may explain the absence of positive sentinel nodes in comparison with our findings. First, SNSCC has the highest rate of occult lymph node metastases among sinonasal malignancies.^18^ Our study exclusively included squamous cell carcinomas, therefore a higher detection rate is plausible. Another potential explanation is the predominance of low T stage tumors in the previous study. However, our data did not demonstrate any correlation between T stage and the likelihood of occult metastasis.

Our study revealed an occult metastasis rate of 9.1%. In the literature, the incidence of occult lymph node metastasis (LNM) in sinonasal carcinomas is reported to be up to 16%, varying significantly by histological subtype.^22,23^ For SCC, the most recent systematic review reports a rate of 12.5% for histologically confirmed occult LNM, with nearly half presenting as pN2 disease.^22^ By contrast, our findings showed only micrometastatic involvement, even after completion of neck dissection. Other authors have reported isolated lymph node recurrence rates of 4.8–7.3% in initially cN0 maxillary sinus carcinoma.^3,8,24^ In other histological subtypes, reported rates are substantially lower.^25–27^ Although most authors agree that END improves locoregional control, its benefit for overall survival remains uncertain.^28^ Consequently, most studies conclude that END is not justified in sinonasal cN0 carcinomas—an assessment also reflected in clinical practice and in interdisciplinary tumor board decisions.^8,17^

We found sentinel nodes predominantly in Levels I and II. This observation is consistent with established patterns of regional lymphatic spread. The lymphatic drainage of the nasal cavity and the paranasal sinuses is organized in highly complex patterns. Anterior regions drain into subdigastric nodes and posterior regions into retropharyngeal nodes, which are the main drainage routes. The drainage of the paranasal sinuses is connected to the nasal cavity collectors.^12,29^ The retropharyngeal nodes being the main drainage of the nose and paranasal sinuses stands in contrast to observations made in terms of metastatic patterns. According to an MRI scan study, clinically suspicious lymph nodes are rarely found in the retropharynx, but more intensively in Level Ib and Level IIa.^30^ In a large retrospective analysis of 299 sinonasal malignancies, confirmed LNM occurred most frequently in Level II (69% of N+ cases), followed by Level I (45%). Retropharyngeal node involvement occurred in only 17% and was mainly associated with large tumors with unclear epicenters.^13^ More recent studies support these findings, although individual cases of Level III involvement have been described.^14^ This is in line with our results, with only 4.5% of patients having sentinel nodes located in Level III. No tracer uptake was detected in retropharyngeal nodes, despite anatomical studies identifying them as primary lymphatic collectors for the nasal cavity and paranasal sinuses.^29^ Interestingly, the above mentioned early study on SNB could detect radioactivity in retropharyngeal nodes in two healthy controls, but not in tumor patients.^12^ When retropharyngeal metastasis is suspected, radiotherapy is typically preferred, as surgical access to these nodes is extremely limited and associated with substantial morbidity.^31^

In terms of surgical performance, we harvested an average of two SNs per patient, which were more than were marked by lymphoscintigraphy, and there were no cases of failed SNB. In well-studied locations, such as the breast or the oral cavity, at least two sentinel nodes are sufficient.^32,33^ Therefore, the diagnostic quality of our study appears adequate and in line with recommendations in the literature.

No complications directly attributable to SNB occurred. Transient facial nerve palsy was observed in three cases, consistent with findings from the aforementioned, retrospective study, although that report provided only final facial nerve status.^21^ Furthermore, both medical professionals and patients assessed the aesthetic outcome as acceptable. The appearance of the scar improved significantly within the first 3 months.

From a health-economic perspective, the introduction of SNB for SNSCC has the potential to reduce overtreatment and associated morbidity. END is an invasive procedure with considerable operative time, longer hospital stays and increased postoperative rehabilitation needs. By contrast, SNB is minimally invasive, can typically be performed during primary tumor surgery, and targets lymphatic basins with significantly lower tissue trauma.^34,35^

Given the relatively low incidence of occult lymph node metastases in sinonasal carcinomas, routine END exposes many patients to unnecessary risk and cost. Although formal cost-effectiveness analyses for sinonasal carcinoma are lacking, data from other head and neck subsites suggest that SNB can reduce overall treatment costs by lowering surgical burden and postoperative complications, while maintaining oncologic safety. For patients with negative SNB a cost reduction of up to 42 % is reported.^36^ These considerations highlight the potential of SNB to improve both resource allocation and patient quality of life.

This study has several limitations. Tumors were solely located in the nasal cavity. Since SNSCC mostly arise in the nasal cavity, the study is lacking data on tumors of the paranasal sinuses. Although all sentinel nodes were successfully identified, the complex anatomy of the sinonasal region may influence tracer distribution, and subtle variations in injection technique or tumor location could affect detectability. Another limitation is the absence of long-term follow-up data, which precludes definitive conclusions regarding the impact of SNB on regional control or survival outcomes. Additionally, micrometastatic disease was the only form of occult metastasis detected, and its biological significance in SNSCC remains insufficiently understood. Comparative studies evaluating recurrence patterns after SNB versus END would be valuable in clarifying this.

Finally, although no SNB-related complications occurred, transient facial nerve palsy was observed, underscoring that even minimally invasive methods can carry procedural risks. Standardized training and adherence to injection and dissection protocols remain essential to minimize variability and ensure reproducibility.

## Conclusions

This study provides prospective, controlled data on feasibility, safety, and oncological outcomes of sentinel node biopsy for patients with sinonasal squamous cell carcinoma. The procedure is considered safe and effective in detecting early lymph node metastases while reducing perioperative morbidity. The results of this study form the basis of a large, randomized confirmatory trial.

## Electronic supplementary material

Below is the link to the electronic supplementary material.

Supplementary file 1 (DOCX 19 kb)
