# Supplementary material for: Sentinel Node Biopsy in the Neck Management of cN0 Sinonasal Squamous Cell Carcinoma: A Multicenter Pilot Trial on Safety and Feasibility
Source: Ann Surg Oncol. 2026 Jun 2;33(12):9981–8. doi: 10.1245/s10434-026-19896-4 (PMC13675268; doi:10.1245/s10434-026-19896-4)
Supplement: Supplementary file 1 [file 10434_2026_19896_MOESM1_ESM.docx]

| **Item** | Vascularity | Pigmentation | Thickness | Relief | Pliability | Surface Area | Overall opinion |
| --- | --- | --- | --- | --- | --- | --- | --- |
| **Mean value postoperatively** | 3.3 | 2.65 | 4.35 | 4.45 | 4.1 | 3.4 | 3.71 |
| **Mean value three months FU** | 2.85 | 3.08 | 3.23 | 4.08 | 3.46 | 3.6 | 2.71 |

Table S1: Mean values of POSAS observer questionnaire.

| **Item** | **Mean value postoperatively** | **Mean value three months FU** |
| --- | --- | --- |
| Has the scar been painful the past few weeks? | 1.75 | 1.69 |
| Has the scar been itching the past few weeks? | 1.95 | 1.85 |
| Is the scar color different from the color of your normal skin at present? | 3.5 | 2.3 |
| Is the stiffness of the scar different from your normal skin at present? | 3.95 | 2.77 |
| Is the thickness of the scar different from your normal skin at present? | 4.45 | 3.9 |
| Is the scar more irregular than your normal skin at present? | 3.65 | 3.1 |
| What is your overall opinion of the scar compared to normal skin? | 3.35 | 3.38 |

Table S2: Mean values of POSAS patient questionnaire.
